# An Online Ethics Curriculum for Short-Term Global Health Experiences: Evaluating a Decade of Use

**DOI:** 10.5334/aogh.3716

**Published:** 2022-08-26

**Authors:** Chelsea E. Modlin, Matthew DeCamp, Michele Barry, Danica Rockney, Jeremy Sugarman

**Affiliations:** 1 Johns Hopkins Berman Institute of Bioethics, Baltimore, MD, US; 2 Department of Infectious Diseases, Johns Hopkins University School of Medicine, Baltimore, MD, US; 3 Center for Bioethics and Humanities, University of Colorado, Aurora, CO, US; 4 Division of General Internal Medicine, University of Colorado, Aurora, CO, US; 5 Center for Innovation in Global Health, Stanford University, Stanford, CA, US; 6 Division of General Internal Medicine, Johns Hopkins University School of Medicine, Baltimore, MD, US

**Keywords:** short-term global health experiences, global health education, ethics education, online education, case-based education, curriculum evaluation

## Abstract

**Background:**

Medical students and early career healthcare professionals commonly participate in short-term experiences in global health (STEGH).

**Objective:**

The authors evaluate the use of a free-to-access, case-based online curriculum addressing ethical issues trainees should consider prior to engaging in STEGH.

**Methods:**

Demographic data and feedback on specific cases were collected from 5,226 respondents accessing the online curriculum between November 1, 2011 and October 31, 2021. Feedback on the curriculum included 5-point Likert scale and open-ended responses. Quantitative data were analyzed using standard descriptive statistics. Qualitative data were independently dual coded and analyzed thematically in NVivo.

**Findings:**

The curriculum reached respondents from 106 countries. Undergraduate (36%) and graduate (38%) respondents included those from several different professional specialties. Less than a quarter of all of respondents, less than half with previous global health experience, and one-third with planned future global health experiences had received prior global health ethics training. Overall, the curriculum was highly rated; respondents felt it provided necessary tools to improve their thought processes, confidence, and behavior when encountering ethical issues during STEGH. Areas for curriculum improvement include balancing case specificity with generalizability.

**Conclusion:**

This curriculum has met a need for accessible introductory global health ethics education and demonstrates successful use of an online platform in case-based ethics learning.

## Introduction

Globalization and a heightening awareness of global health disparities has fueled an extraordinary increase in global health interest among medical students and early-career healthcare professionals [1,2,3]. In response to the associated demand for global health education, there has been a significant increase in the number of global health programs at academic institutions at the undergraduate, graduate, and post-graduate level within the past two decades, especially within the United States [1,4,5]. These programs often partner with international institutions to sponsor students and faculty participation in short-term experiences in global health (STEGH) [6,7].

Engaging in global health education and international STEGH positively impacts early career development. Among medical students, involvement in a global health program is associated with enhanced medical knowledge [8], communication skills [9], and cultural sensitivity [10]. These students are more likely to pursue careers working with underserved populations and promote health equity in clinical practice [11,12].

But there are also potential drawbacks. STEGH can involve ethical issues and concerns that arise from unfamiliar approaches to medical practice, different cultural norms, and lack of access to resources [13,14]. These concerns are related to both clinical practice (e.g., limited material resources, end-of-life care) and research (e.g., informed consent, equity within international research collaborations) [15]. Left unaddressed, such ethical concerns can result in emotional and moral distress for both visiting participants and host preceptors [16], inappropriate allocation of limited resources [10,17], and inadvertent worsening global health inequities [18].

To address these issues, various guidelines have been developed [19]. One set of best practice guidelines was created through an international effort of the Working Group on Ethics Guidelines for Global Health Training (WEIGHT) [20]. These recommendations specify the need for focused pre-departure curricula on the logistical, cultural, and ethical components of STEGH. While ideally tailored to specific international sites and sponsoring programs, an introductory ethics curriculum targeted towards participants was needed.

In 2011, and building on WEIGHT, we developed an introductory ethics curriculum that is freely-available online, titled ‘Ethical Challenges in Short-Term Global Health Training’ (http://ethicsandglobalhealth.org). In this article, we describe the users of the curriculum over the past decade and assess its ability to reach the intended target audience of student and early-career clinicians and clinical researchers. We additionally summarize the curriculum’s reception and respondent-generated feedback to inform its future development and support further use of online platforms for global health ethics education.

## Method

### Curriculum Development

The design and original implementation of the curriculum has been previously described [21]. Briefly, cases were solicited from trainees and program leaders within global health programs, including international members from WEIGHT and members of the Consortium of Universities for Global Health (CUGH). A purposive strategy was applied to select and develop ten scenarios with an emphasis on major ethical themes in STEGH. A brief audio vignette with photos, multiple-choice questions, and a resource page were drafted for each case. The curriculum was formally launched in November 2011.

### Data Collection

Between November 1, 2011 and October 31, 2021, data from trainees accessing the curriculum were requested anonymously and administered via Survey Monkey™. Completing surveys was not mandatory in order to use the curriculum. The Johns Hopkins Medicine IRB [NA_00064935] and the Colorado Multiple Institutional Review Board [21–4866] determined that this work was exempt from review. A demographic survey (**Supplementary Figure 1**) was accessible from various points within the website and included basic demographic information, prior experiences in global health, and prior ethics training. Following the completion of each case, trainees were additionally asked to complete a case-specific survey (**Supplementary Figure 2**) using 5-point Likert scales (from “strongly disagree” to “strongly agree”) and provide open-ended feedback particular to each case.

Web statistics, including hits, pages, and visits, were provided by the web host; however, these results proved highly unreliable. For example, there was no way to differentiate between a visit from an actual person versus web crawlers and other automated programs, which greatly increased measured traffic to the site. The web host does not provide metrics of user interactivity with specific site components such as playing the audio vignette or answering the embedded multiple-choice questions used to engage the learner with the cases. Therefore, our analysis was conducted based on demographic and survey response data collected from actual respondents, which under-represents total use due to the voluntary nature of the survey.

### Data Analysis

A mixed-methods approach was used. Quantitative data were analyzed using standard descriptive statistics in Microsoft Excel. Respondent citizenship mapping was conducted using Plotly Chart Studio (Plotly Technologies Inc, released 2015). Responses to open-ended feedback questions were analyzed qualitatively using NVivo 1.0 (QRS International, released 2020). Feedback was coded as positive, negative or neutral and additionally reviewed for constructive feedback regarding the theme, content, or structure of each case and for general usability of the web platform. Thematic coding was done independently by two coders and discrepancies were resolved by consensus.

## Results

The demographic survey was completed by 5,226 respondents with a gradual increase in number of respondents per month. Feedback on individual cases indicates a higher number of total respondents (e.g., with 8,998 responses to Case 1—Developing Cultural Understanding, since all respondents did not complete the demographic survey). The number of respondents providing feedback on individual cases varied; Case 7—Recognizing Burdens elicited the fewest responses (n = 5,073).

**Figure 1:**
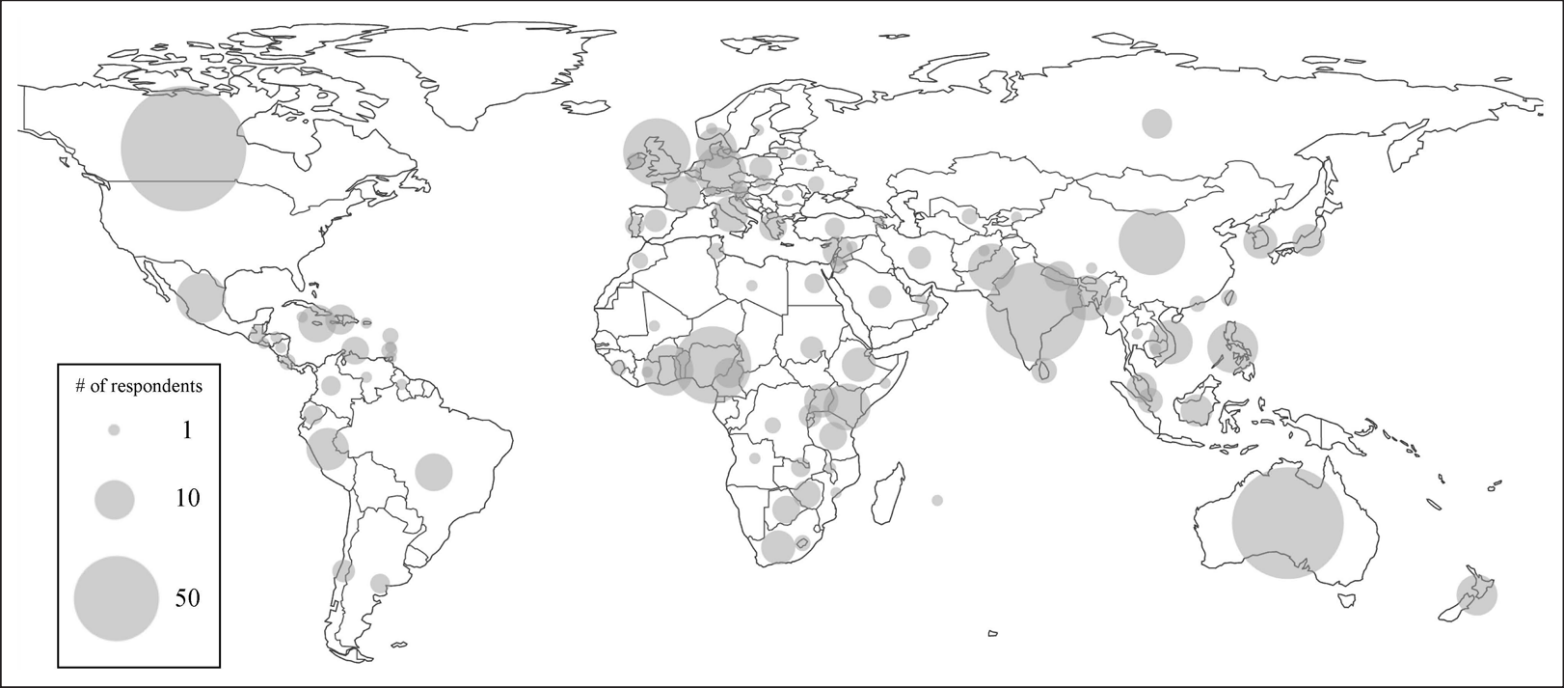
Bubble map depicting the geographic distribution of respondents by reported citizenship excluding United States. Size of the bubble is proportional to the number of respondents from each country.

Respondent demographic characteristics are summarized in Table 1. Most were female (74%, 3,834/5,208) and within the 18–29 year of age group (66%, 3,371/5,113). The majority were United States citizens; however, 18% (936/5,186) reported non-US citizenships, the most frequent of which were Canada, Australia, and India. A total of 106 discrete non-US citizenships were reported. A world map depicting non-US respondent citizenship is available in Figure 1. About one-third of respondents were enrolled in a bachelor degree-granting program and another third were enrolled in masters or doctorate-level training. Medicine (44%, 2,294/5,140), nursing (21%, 1,098/5,140), and public health (10%, 540/5,140) were the most frequently reported fields of education or profession.

**Table 1:** Demographic data from survey respondents from November 1, 2011 through October 31, 2021.


CHARACTERISTIC	CATEGORY RESPONDENTS^a^, NO. (%)	RESPONDENTS, NO. (%)

Female	5,208 (99.7)	3,834 (73.6)

Age	5,113 (97.8)	

18–29		3,371 (65.7)

30–39		856 (16.7)

40–49		428 (8.4)

50–59		316 (6.2)

60 and above		142 (2.8)

Ethnicity	5,190 (99.3)	

American Indian or Alaska Native		31 (0.6)

Asian		859 (16.6)

Black or African-American		525 (10.1)

Native Hawaiian or Other Pacific Islander		23 (0.4)

White		3,177 (61.2)

Mixed		328 (6.3)

Other		247 (4.8)

Hispanic Origin	5,170 (98.9)	653 (12.6)

United States Citizens	5,186 (99.2)	4,250 (82.0)

Current Degree Program	5,032 (96.3)	

High School		7 (0.01)

Bachelor’s Degree		1,833 (36.4)

Master’s Degree		707 (14.1)

Doctorate Degree		1,208 (24.0)

Currently Practicing		977 (19.4)

Other		300 (0.6)

Primary Field/Vocation	5,140 (98.4)	

Basic Science		114 (2.2)

Engineering		35 (0.7)

Health Policy		79 (1.5)

International Aid/Development		90 (1.7)

Medicine		2,294 (44.1)

Nursing		1,098 (21.1)

Pharmacy		56 (1.1)

Physical Therapy		65 (1.2)

Physician’s Assistant		151 (2.9)

Public Health		540 (10.4)

Social Sciences		197 (3.8)

Other		484 (9.3)


^a^ Number of total respondents to each category of characteristics out of overall number of survey respondents (n = 5,226). Respondents do not have to answer all questions.

Respondents were additionally asked about previous and planned travel for STEGH (Table 2). Only one-third reported previous travel, with half of those experiences being less than 4 weeks long in duration. Among those with previous travel, 43% (768/1,779) reported previous global health ethics training compared to only 13% (429/3,382) of respondents with no previous travel. An increase in the proportion of respondents with ethics training was seen with more previous trips reported (χ^2^ = 41, p-value < 0.001). Of the 45% (2,326/5,126) of respondents planning future travel, 35% (810/2,326) had prior ethics training; 39% (902/2,326) indicated this curriculum was the only ethics training they would receive; an additional 35% (817/2,326) were unsure if any further ethics training was planned. The complete curriculum was required for 52% (2,582/4,996) of respondents as a part of their program.

**Table 2:** Previous and planned global health experience and global health ethics training from survey respondents from November 1, 2011 through October 31, 2021.


CHARACTERISTIC	CATEGORY RESPONDENTS^a^, NO. (%)	RESPONDENTS, NO. (%)

Previous Travel	5,190 (99.3)	1,808 (34.8)

Duration of Previous Travel	1,785 (34.2)	

Less than 4 weeks		922 (51.7)

4–8 weeks		417 (23.4)

8–12 weeks		130 (7.3)

Greater than 12 weeks		316 (17.7)

Location of Previous Travel	1,783 (34.1)	

Africa		746 (41.8)

Americas		1,051 (58.9)

Eastern Mediterranean		79 (4.4)

Europe		186 (10.4)

Southeast Asia		428 (24.0)

Western Pacific		84 (4.7)

Other		211 (11.8)

Future Travel Planned	5,126 (98.1)	2,326 (45.4)

Duration of Future Travel	2,319 (44.4)	

Less than 4 weeks		1,027 (44.3)

4–8 weeks		928 (40.0)

8–12 weeks		127 (5.5)

Greater than 12 weeks		237 (10.2)

Location of Future Travel	2,354 (45.0)	

Africa		800 (34.0)

Americas		849 (36.1)

Eastern Mediterranean		32 (1.4)

Europe		85 (3.6)

Southeast Asia		310 (13.2)

Western Pacific		46 (2.0)

Other		232 (9.9)

Previous Ethics Training	5,116 (97.9)	1,201 (23.5)

Among Previous Travel Abroad	1,779 (34.0)	768 (43.2)

1–2 trips	1,007 (19.3)	372 (36.9)

3–5 trips	393 (7.5)	187 (47.6)

>5 trips	379 (7.3)	209 (55.1)

This Curriculum is Only Training	5,003 (95.7)	

Yes		1,603 (32.0)

No		1,236 (24.7)

Do Not Know		2,164 (43.2)

This Curriculum is Required	4,996 (95.6)	2,582 (51.7)


^a^ Number of total respondents to each category of characteristics out of overall number of survey respondents (n = 5,226). Respondents do not have to answer all questions.

**Figure 2:**
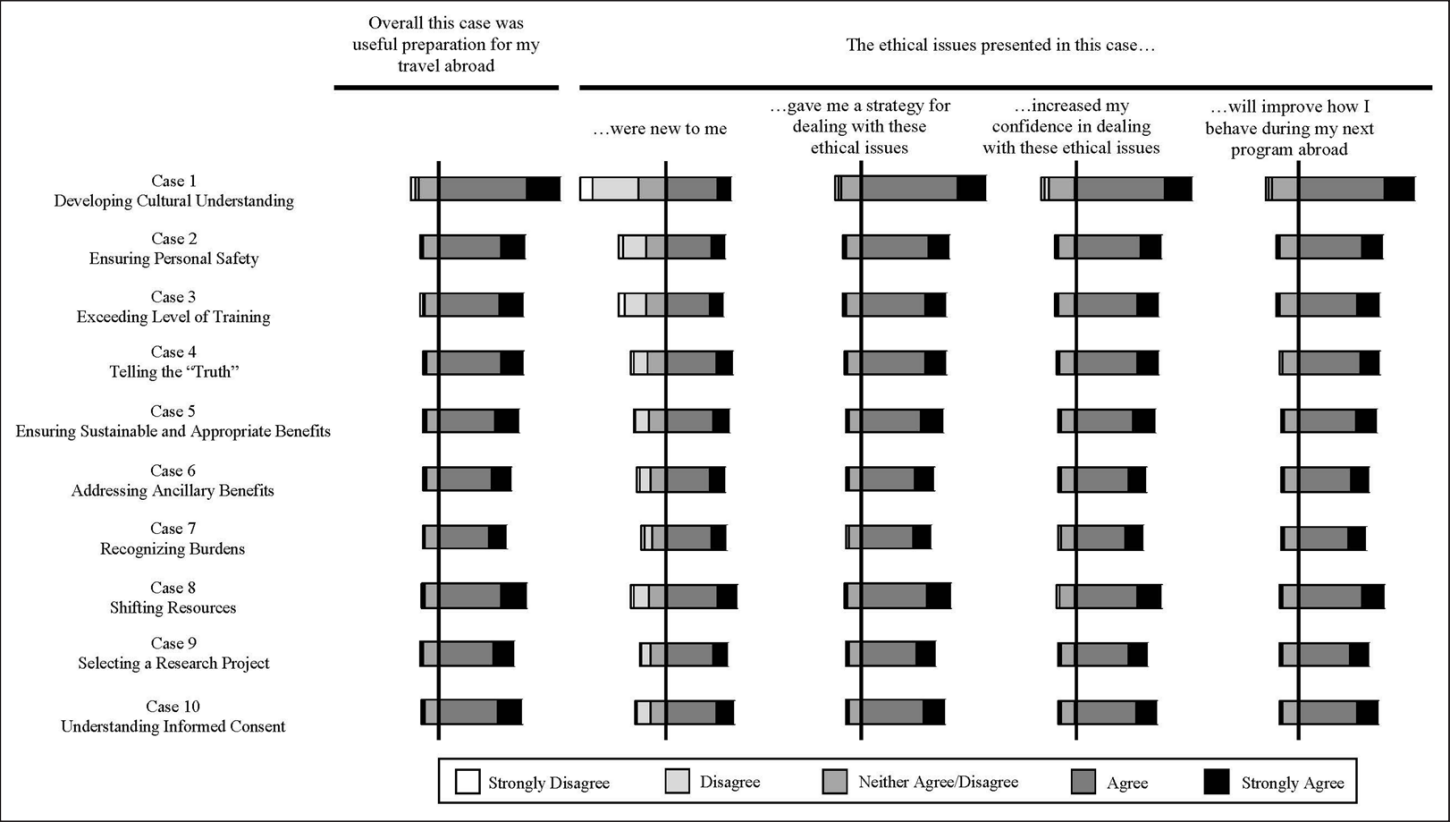
Diverging stacked bar representation of Likert responses to questions about each of the cases included in the curriculum. Vertical line represents opinion cutoff between favorable (agree, strongly agree) and unfavorable/neutral (disagree, strongly disagree, neither agree/disagree).

All ten cases were reviewed favorably (Figure 2). With the exception of case 1 (Developing Cultural Understanding), each was also rated as presenting a new concept. Respondents indicated that all of the cases provided tools to improve their thought process, confidence, and behavior when encountering ethical issues during STEGH.

Qualitative analysis of open-ended feedback for each case revealed a similarly positive reception. Overall, respondents found the curriculum helpful and thought-provoking. A summary of constructive feedback on the cases’ themes and content are outlined in Table 3. In some of the cases, feedback indicated that details within the case raised concern for stereotyping depicted host communities. In contrast, others requested to have more specific information about the location in which the case was set. In particular, attention was drawn to potential unintended gender and racial stereotyping in the depiction of medical trainees. This included highlighting that in one case a female medical student was advised to change her behavior to adhere to cultural norms while not specifying that a male student be asked to do the same. Several cases concluded with a recommendation to discuss concerns and questions with local STEGH supervisors, but respondents found this to be unsatisfactory and redundant across cases. Rather, there was an expressed preference for more concrete solutions to the cases presented. In addition, several respondents indicated they either would not have an in-country supervisor or would be uncomfortable with bringing up concerns about ethical or cultural conflicts. Many respondents expressed an interest in moving beyond introductory material with coverage of more ethically and socially complex topics, and several indicated that their previous experiences rendered the curriculum too simple. Nonetheless, others appealed for the inclusion of even more introductory material, citing a lack of knowledge of some basic ethics and global health terminology that was used in the curriculum. For example, one respondent indicated that they were not aware of the definitions of terms ‘beneficence’ and ‘distributive justice’ while several others had not heard of the ‘brain drain’ phenomenon in global health. While the original cases were designed primarily for clinicians, the use of the curriculum by a broader range of trainees from other professional backgrounds was associated with an interest in seeing cases relevant to other areas of global health, including public health, international aid development, and occupational and physical therapy. With progression of website design technology over the 10 years the curriculum has been active, updates to the website formatting were recommended, including revision of the audio vignettes to the use of actors and live-action video within the case presentations.

**Table 3:** Thematic coding categories and examples.


CODE	DESCRIPTION	EXAMPLE(S)

Positive		

Comprehension	Expression of new material understanding based on case content.	“This case helped me increase awareness of cultural differences in different countries.”

		“’Anticipate, ask act’ was a helpful strategy for the person featured in the vignette to react, and it will be helpful for me too.”

Novelty	Expression that case content or theme were new to the respondent.	“A lot of these issues were things I have never thought about and am thankful for learning about.”

		“I was not aware of some of the procedures you should consider…and thought they are of great help in understanding consent from other cultures.”

Relevance	Content reported to be relevant to respondent interests, travel plans, or educational goals.	“…excellently illustrated a common issue that many travelers may not fully comprehend when attempting research. It helped to frame the issue appropriately, and provide effective strategies for potential issues.”

		“I actually thought this was really relevant and would not have thought about these issues.”

Succinct	Cases and content reported as concise, succinct, etc.	“Brief and to the point. It got the message across in just a few minutes. Wouldn’t change it.”

Negative		

Basic	Content and themes considered too basic or rudimentary to be of educational benefit.	“Substantively it was too basic.”

		“This case is too easy to be of any benefit to anyone.”

Lack of Relevance	Content lacks relevance to the travel activities or interest of the respondent.	“Case was fine. I will not be conducting research, so feels less relevant.”

		“Not relevant to my plans.”

Case Content		

Actionability	Statement and degree to which respondents felt case theme was actionable within their learning or experience.	“I like the practical vignette; such a situation could arise in several parts of the world and it gave me tools to better prepare and deal with if encountered in the future.” “The topic is important and perhaps broad but I did not glean any insightful next steps or action items should I find myself if any of the situations discussed in the case.”

Ambiguity	Unclear or contradictory content within the case.	“The topic is important and perhaps broad but I did not glean any insightful next steps or action items should I find myself if any of the situations discussed in the case.”

		“This case was not well elaborated…the representation of the issue needs to be better explained. I found it somewhat confusing.”

		“I have no idea how distributive justice has anything to do with donating property. That should be explained better.”

Burden of Responsibility	Comment on distribution of responsibility and/or accountability among trainees and institutions.	“I feel that the program sending trainees abroad should have a more direct role in these situations. Higher ups in the programs in developed countries should be talking with their colleagues about situations like this before hand. Trainees are relatively disempowered on the ground in such situations.”

		“I knew my host institution would be sponsoring meals for us and I was concerned about causing burden. Thinking about this ahead of time helps and I hope that in speaking with my advisers, we ensure an equitable balance between giving and receiving.”

Complexity	Comment of the level of complexity of the case material.	“I feel that this module may be too simple. Maybe more detail and/or complex cases would make it more of a challenging assignment.”

		“The case as presented was over simplified version of this complex issue…and did not necessarily deal with all the issues on the most practical or realistic way.”

Cultural Competency	Comment about need for/lack of/appropriate level of cultural competency or awareness within the case of for people in similar situations.	“It was a great example of telling the truth across different cultures. It just emphasizes the importance of understanding local culture before working there.”

		“Cultural competence should be a requirement for all medical student traveling to study in another country. This mandate would better prepare students on how to handle critical situation that would also prevent them from being looked down upon by the locals.”

Gender Roles	Participant points out specifics within case content regarding character gender or gender norms.	“I disliked the fact that this focused on the females as the “violators” of the cultural norms as it is also equally the responsibility of the male [characters].”

		“Navigating different expectations for male and female behavior in the host culture is part of this exercise, however the questions focused only on the conduct of the female visiting students. [Male character] is also an ambassador of the training program and the larger school community, and the training questions could explore his role as well.”

Previous Exposure to Topic	Respondent comment that topic of the case has been encountered or experienced previously.	“The only reason I personally did not find it beneficial is because I have had experience navigating different cultures therefore it was not new to me.”

		“This exact issue has come up during a past experience, so I was pleased to see it presented as part of this course.”

		“Even though I do have a great deal of experience working overseas and with students, this reinforced and reminded me of how important it is to practice what we know to be safe measures. It is all too easy to forget these when you are in resource poor areas but that is no excuse.”

Resource Limitations	Respondent comment regarding location resource or capacity limitations.	“It would be nice to see a discussion about why the clinic is so poorly supplied – and the ways in which people living in other countries with more resources can improve access to essential medicines such as antibiotics.”

		“This specific case didn’t give many actual recommendations or examples of how to handle resources in resource-scarce settings. More information regarding program sustainability would also be useful.”

Stakeholder Input	Comment on need for/example of/case content regarding stakeholder evaluation or eliciting community input.	“I really liked the answers that involved asking for the locals’ feedback. It demonstrates thoughtfulness and care.”

		“The idea of involving the local community in decision making is great, but making this happen is often quite challenging. Who makes up the ‘local representation’, how is the decision on who to include in that process made?”

User Disagreement	Respondent expresses disagreement with a detail, theme, or conclusion from the case content.	“I think it is unrealistic to say that you can always temporarily excuse yourself from a patient encounter to consult a local advisor. Some decisions have to be made on the spot and local advisors aren’t always available.”

		“I disagree about the discussion whether the student should explain that she is “not a doctor.” I feel that that [sic] term in some countries is used as health care workers…as such would be more meaningful in describing her role than student.”

Suggestions	Respondent provides specific suggestion for improvement or alteration of the case content or theme.	“Please elaborate the scenarios by including other cultural differences, e.g. dress code, mannerisms, tone and volume of conversations (Americans tend to be more boisterous than European and Asian and it is often misunderstood for arrogance and rudeness).”

		“I was most interested in this case because I am a surgeon who has worked extensively in Africa and still struggle with the concept of consent. I wish you would add on a module dealing with consent only done by husbands or head of families for operations such as amputation, tubal ligation, C section this is often the case.”

		“I would appreciate recommendations for LGBTQI+ individuals travelling to areas where non-heterosexuality is not “culturally appropriate” or even illegal. At what point does adhering to cultural norms violate personal expression and self-identity? How should personal safety be handled?”


## Discussion

Over the past decade, the ‘Ethical Challenges in Short-Term Global Health Training’ online curriculum has been accessed by an increasing number of geographically and professionally diverse users. Feedback about the curriculum has been overwhelmingly positive. Our findings demonstrate that this curriculum provides an important accessible online introduction to ethical issues in STEGH. Nevertheless, there are some limitations and lessons learned in the creation, applicability, and sustainability of this online case-based approach to ethics and global health.

The need for short-term global health ethics training is evident. Despite several proposed curricula frameworks [22,23], consensus guidelines [20,24], and recognition of ethics as a global health competency [25], the development of comprehensive global health educational competencies regarding ethics and professional or social responsibility is still in its earliest stages [26]. Ethics topics are commonly treated as a whole and are combined into one competency or grouped with professionalism for global health work. This is seen in the domains of clinical medicine [25,27], nursing [28], research [29], and public health [30]. We found that less than half of respondents with previous STEGH had prior ethics training, and the likelihood of having had such training correlated with the number of previous trips. An even lower proportion of respondents with planned future travel reported prior ethics training. This is in direct contradiction to preferences expressed by international host preceptors that visiting trainees should be required to receive dedicated ethics training prior to traveling abroad [31].

Case-based learning is familiar to medical trainees for effective teaching in both medicine [32] and bioethics [33,34]. For example, medical students enrolled in a case- and discussion-based ethics pre-departure STEGH curriculum reported improved identification and processing of a broad range of ethical conflicts during post-trip debriefing [35]. Cases are also the foundation of the Simulation for Global Health Away Rotations (SUGAR) curriculum [36], which offers structured prompts and facilitator training to guide medical trainees through common emotional reactions encountered during STEGH. Experiential immersion is taken even further by the ESIGHT program [37] and Health, Equity, and Leadership (HEAL) Initiative [38] at the University of California, San Francisco, which uses interactive, simulated encounters featuring actors to recreate ethically and emotionally distressing scenarios trainees may confront. While such experiential learning is desirable, access to such resources is limited for all preparing for STEGH. Therefore, there remains a need for more widely available case-based learning in ethics and global health. The accessibility, adaptability, and searchability of online platforms can help meet this need.

This curriculum represents one of the few long-standing and easily accessible online global health ethics curricula. We have previously reviewed other short-term global health ethics curricula [21], including the Unite for Sight Volunteer Ethics and Professionalism Course (available at https://www.uniteforsight.org/global-health-course/) and the Ethics of International Engagement and Service Learning (EIESL) Guidebook (available at http://ethicsofisl.ubc.ca), both of which incorporate ethics into learning modules on professionalism. More recently available is The Practitioner’s Guide to Global Health (PGGH) [39], which similarly offers free, asynchronous preparation tools for medical trainees planning STEGH, with particular attention to logistics and personal safety. Unlike our curriculum, these other online curricula place less emphasis on case-based learning.

Ethics education using online platforms has been known to have both benefits and drawbacks [40]. Advantages include flexibility to fit individual schedules and different learner types. For example, we provide audiovisual vignettes for audio learners while transcriptions are available for those who learn better by reading. Limitations to online ethics education include difficulty achieving learner comprehension of more complicated ethical concepts [41]. Application of theoretical knowledge to real-world cases with post-case assessment is one method to overcome this challenge, which was used in our curriculum. Overall, feedback from respondents suggested comprehension of key learning objectives and durability of the curriculum over time. The dissemination of the curriculum online also likely contributes to its accessibility to an international user base.

We encountered a difficult balance in attempting to satisfy the level of detail needed to make a case effective. On one hand there was a desire to present scenarios more generally, both to protect individuals originally involved—given they were all based on real cases—and to make each scenario applicable to a larger audience. However, some respondents felt this generalization detracted from the effectiveness of the modules and decreased relevance to their educational objectives. On the other hand, some raised concerns that cases ran the risk of stereotyping local clinicians, populations, and cultures. This tension between generalizability and specificity requires careful selection and revision of current and future cases.

The curriculum has met a critical need for ethics training. One third of total respondents, and the majority preparing for STEGH, indicated that either this curriculum was the only source of ethics training they would receive or that they were unsure if any further training was planned. However, specific ethical issues faced by an individual student or program cohort are going to be unique to both host location and sponsoring institution. While several case themes can be somewhat generalized across host locations, for example, unintended burdens to host programs, it could be problematic if this curriculum is the only training available to trainees. When required as part of a pre-departure curriculum, as it was for over half of respondents in our analysis, these online cases should ideally be used as an adjunctive teaching tool that is further tailored to the needs and specifics of each global health program and host site. We also found it troublesome that some respondents indicated they either would not have a host supervisor or were not comfortable approaching program leadership to discuss ethical and cultural conflicts during their global health experience. This correlates with findings from Peluso et al., who found only 21% of medical students participating in STEGH sought local provider input on witnessed ethical conflicts [35]. Inclusion of direct, appropriate supervision and mentorship is an important part of equitable STEGH partnerships, and should not be overlooked [20].

The curriculum’s reach extended well beyond what was the originally intended audience of medical trainees, most often from high-income settings like the United States, preparing to travel internationally to more resource-limited areas. In actuality, the curriculum was accessed by users in both high-income (i.e., global North) as well as low- and low-/middle-income countries (i.e. global South). This suggests a general interest in the topic and reinforces the bidirectionality of these ethical issues between the global North and South [42] in academic collaborations. It also provides the unique opportunity to elicit and incorporate specific feedback from local stakeholders for cases with distinct geographic or cultural learning objectives. Additionally, use of the curriculum extended beyond medical students and clinicians, incorporating respondents with a wide range of professional backgrounds, including nursing, public health, social sciences, and basic sciences, among others. This diversity suggests a broader interest about ethical issues seen in STEGH and a need for further investigation into specific concerns within these professional areas.

Our quantitative and qualitative analyses yield important areas in which the curriculum could be improved and areas for lessons on global health ethics curricula generally. First, our evaluation suggests that current educational material could be refined to include actors and topics reflective of the diverse professional trainees accessing the curriculum. Even if ethics concepts cross disciplines, tailored training is likely to be more effective. Second, some cases addressed less ‘new’ topics (e.g., cultural understanding), perhaps because trainees receive education on these topics in other fora. Third, the world has changed in the decade since our curriculum launched, with greater attention to structural racism, gender inequality, and other issues of equity. There is a need to revisit our cases, refining them through these lenses or creating new ones that address these topics. Fourth, in the era of the COVID-19 pandemic, inclusion of ethical issues around international epidemic health response would be highly relevant.

Regarding global health ethics curricula generally, our findings suggest that they must navigate a delicate balance between the context- and location-specific ethics advice and more generalized depictions. Unintentional harm can also occur from overgeneralizing and inadvertently stereotyping people, places, and culture, about which curriculum developers need to be aware. In addition, given the existence of multiple global health ethics curricula for STEGH, there is a need to compare their relative effectiveness and to rigorously evaluate them with behavioral and partnership-based outcomes, not just their perceived effectiveness or self-reported outcomes (such as confidence). Finally, the growing movement to “decolonize” global health should also apply to global health ethics, creating a need to incorporate more content and case analyses not just contributed by, but also led by low- and middle-income partners.

Despite the importance of our findings, there are several limitations to our assessment that should be considered in interpreting the results. Since the original launch of the curriculum there was a change in the mechanism by which our website analytic software collected user data, resulting in an inability to estimate the true number of human users accessing the site. While demographic information and feedback was requested from individual users, this was optional, which results in an underestimated number of site users, and likely subjected our analysis to a degree of ascertainment bias. This curriculum is also only available in English, limiting its accessibility to only English-reading users.

We conclude that the Ethical Challenges in Short-Term Global Health Training curriculum has reached an international and professionally diverse usership over the past 10 years since its launch. Its structure, online format, and case content is overall well-received and helpful for trainees new to ethical issues within global health training. However, the curriculum is introductory and alone is not sufficient for comprehensive global health ethics predeparture training. Our future plan is to update its content, interface, and usability, and seek ways to integrate it into more comprehensive training programs and evaluate its application on the ethics competency as well as behavior of trainees during STEGH.

## Additional Files

The additional files for this article can be found as follows:

10.5334/aogh.3716.s1 Supplementary Figure 1. Open user anonymous demographic survey.

10.5334/aogh.3716.s2 Supplementary Figure 2. Case-specific feedback survey questions.
