# Supplementary material for: An Online Ethics Curriculum for Short-Term Global Health Experiences: Evaluating a Decade of Use
Source: Ann Glob Health. 2022 Aug 26;88(1):74. doi: 10.5334/aogh.3716 (PMC9414809; doi:10.5334/aogh.3716)
Supplement: Supplementary Figure 1. — Open user anonymous demographic survey. [file agh-88-1-3716-s1.pdf]

Supplementary Figure 1. Open user anonymous demographic survey.

About You

1. Are you male or female?

- ◯ Male
- ◯ Female

2. How old are you?

[ ]

3. What is your race/ethnicity?

- ◯ American Indian or Alaska Native
- ◯ Asian
- ◯ Black or African American
- ◯ Native Hawaiian or Other Pacific Islander
- ◯ White
- ◯ Mixed
- ◯ Other (please specify)

[ ]

4. Are you of Hispanic, Latino, or Spanish Origin?

- No
- Yes – Mexican or Mexican-American, Chicano
- Yes – Puerto Rican
- Yes – Cuban
- Yes - Another (please specify)

5. What is your nation of citizenship?

- United States
- Other (please specify)

6. What level of education have you achieved up till now? (Select the highest achieved)

- High School
- Vocational School
- Bachelor’s Degree/University
- Master’s Degree
- Doctorate
- None of the above

7. If you are currently in a degree granting program, what degree are you pursuing?

- High School
- Bachelor’s Degree/University
- Master’s Degree
- Doctorate
- Not applicable (currently practicing in my field)
- Other (please specify)

8. What specialty or field do you consider your main vocation?

- Basic Science
- Engineering
- Health Policy
- International Development/Aid
- Medicine
- Nursing
- Pharmacy
- Physical Therapy
- Physician’s Assistant
- Public Health
- Social Sciences

Other (please specify)

<u>About Your Prior Experience in Global Health</u>

9. Have you been abroad previously for global health training or service?

- Yes
- No

10. How many times have you been abroad previously for global health training or service purposes? (If 0, skip to question 13.)

- 0
- 1-2
- 3-5
- More than 5

11. On average, how long were these prior trips abroad for global health training or service purposes?

- Less than 4 weeks
- 4-8 weeks
- 8-12 weeks
- Greater than 12 weeks

12. In what regions have you previously traveled for global health training or service purposes? Choose

all that apply.

- [ ] Africa
- [ ] Americas
- [ ] Eastern Mediterranean
- [ ] Europe
- [ ] Southeast Asia
- [ ] Western Pacific
- [ ] Other (please specify)

<u>About Your Next Global Health Training or Service Program</u>

13. Do you have a future short-term global health training or service program planned? (If "No," skip to question 16.)

- ○ Yes
- ○ No

<u>About Your Next Global Health Training or Service Program</u>

14. Where will this next training or service program be? Choose all that apply.

- ○ Africa
- ○ Americas
- ○ Eastern Mediterranean

- Europe
- Southeast Asia
- Western Pacific
- Other (please specify)

15. How long will this future training or service program be?

- Less than 4 weeks
- 4-8 weeks
- 8-12 weeks
- Greater than 12 weeks

About Your Prior Experience in Global Health Ethics

16. Have you had prior global health ethics training?

- Yes
- No

17. Was this prior ethics training directly relevant to short-term training or service abroad?

- Yes
- No

About this Introductory Curriculum

18. Will completion of this curriculum be your only ethics training prior to your next short-term training or service program abroad?

- Yes
- No
- Don't know - unsure what program is planning
- Don't know - no program planned

19. Is completion of this curriculum required by your training or service program?

- Yes
- No
- Not applicable - no program planned

20. How did you learn about this curriculum? Choose all that apply.

- Referral from a friend or colleague
- Through my training program
- Web search
- Other (please specify)
