# Supplementary material for: An Online Ethics Curriculum for Short-Term Global Health Experiences: Evaluating a Decade of Use
Source: Ann Glob Health. 2022 Aug 26;88(1):74. doi: 10.5334/aogh.3716 (PMC9414809; doi:10.5334/aogh.3716)
Supplement: Supplementary Figure 2. — Case-specific feedback survey questions. [file agh-88-1-3716-s2.pdf]

Supplementary Figure 2. Case-specific feedback survey questions.

1. Overall this case was useful preparation for my training abroad.

○ Strongly Disagree	○ Disagree	○ Neither agree/disagree	○ Agree	○ Strongly Agree

2. The ethical issues presented in this case...

	**Strongly Disagree**	**Disagree**	**Neither agree/disagree**	**Agree**	**Strongly Agree**
...were new to me.	○	○	○	○	○
...gave me a strategy for dealing with these ethical issues.	○	○	○	○	○
...increased my confidence in dealing with these ethical issues.	○	○	○	○	○
...will improve how I behave during my next training program abroad.	○	○	○	○	○

3. Why did you do this case?

○ Requirement of my program

○ Thought it would be of personal benefit

○ Both

4. Do you have any recommendations for changing this case?
Comment on structural (e.g., web design, font, video performance, etc.) and/or substantive (e.g., level of detail, concreteness of strategies, representation of issue) concerns.

5. Would you recommend this case to a friend?

Yes No
